# Impact of hyperkalaemia definition on incidence assessment: implications for epidemiological research based on a large cohort study in newly diagnosed heart failure patients in primary care

**DOI:** 10.1186/s12875-016-0448-5

**Published:** 2016-05-04

**Authors:** Mar Martín-Pérez, Ana Ruigómez, Alexander Michel, Luis A. García Rodríguez

**Affiliations:** Spanish Centre for Pharmacoepidemiologic Research (CEIFE), Madrid, Spain; Epidemiology, Bayer Pharma AG, 13342 Berlin, Germany

**Keywords:** Hyperkalaemia, Heart failure, Incidence, Cohort study, Primary care

## Abstract

**Background:**

Various definitions of hyperkalaemia have been used in clinical research, and data from routine clinical practice on its incidence are sparse. We aimed to establish the incidence of hyperkalaemia in patients with newly diagnosed heart failure in the UK general population using different definitions for the condition.

**Methods:**

We conducted a large retrospective cohort study using data from The Health Improvement Network primary care database. Patients with newly diagnosed heart failure (*N* = 19,194) were identified and followed until the first occurrence of hyperkalaemia. Different serum potassium (K^+^) thresholds were evaluated as possible definitions for hyperkalaemia, and incidence rates (IRs) calculated using a final operational definition both overall and among patient sub-groups.

**Results:**

IRs of hyperkalaemia ranged from 0.92–7.93 per 100 person-years according to the definition. Based on considerable differences in the serum K^+^ normal range used between practices, 2176 (11.3 %) individuals were identified with a record of hyperkalaemia using our operational definition of a proportional increase of ≥10 % above the upper bound of the normal range: IR 2.90 per 100 person-years (95 % CI 2.78–3.02) over a mean follow-up of 3.91 years. Incidence rates were higher in older patients, and in those with diabetes or renal impairment.

**Conclusions:**

Hyperkalaemia is a common finding in heart failure patients in primary care, but its incidence can vary nearly ten-fold depending on its definition. Since assessment of hyperkalaemia risk is essential for therapeutic decision making in heart failure patients, this finding warrants consideration in future epidemiological studies.

## Background

Hyperkalaemia is a common condition in patients with heart failure with most cases either asymptomatic or associated with mild, non-specific symptoms, yet more severe clinical manifestations can occur without warning [1]. Population based time-series analyses have demonstrated significant increases in rates of hospitalisation and mortality from hyperkalaemia in real-world clinical practice, correlating with the more widespread use of medications inhibiting the renin-angiotensin-aldosterone system (RAAS) [2, 3]. These medications – either alone or in combination – are effective therapies for patients with heart failure, yet it is important to assess the risk of hyperkalaemia before initiating treatment because many of these patients already have impaired potassium excretion placing them at increased risk of hyperkalaemia, in particular the elderly and those with renal impairment or diabetes [4–6]. Incorporating the risk of hyperkalaemia into therapeutic decision making in heart failure patients depends upon having a precise definition for an episode of the condition, yet at present there is no consensus regarding the most appropriate threshold of serum potassium (K^+^) to apply – various thresholds [7–11] or other operational definitions [12] for hyperkalaemia have been used in previous studies.

Current data on the incidence of hyperkalaemia in patients with heart failure come mainly from clinical trial populations treated with RAAS antagonists [7, 12, 13], which may not reflect the actual burden of hyperkalaemia in clinical practice. Patients entering clinical trials are selected carefully, excluding those who are considered to be at highest risk for potential adverse reactions with use of the study medication. Population-based data from routine clinical practice focusing on the incidence of hyperkalaemia are sparse, yet are potentially attainable through the use of large population-based databases of anonymized electronic medical records (EMRs). The Health Improvement Network (THIN) [14] is one of several such databases arising from general practices throughout the United Kingdom (UK), which are increasingly being used for pharmacoepidemiological research. They enable long-term follow-up of observational cohorts, and are able to provide large samples that are often representative of the target population. This study aimed to i) estimate the incidence of hyperkalaemia in a large population-based cohort of incident heart failure patients in THIN using different definitions of the outcome, and ii) using a final operational definition for hyperkalaemia, estimate the incidence of hyperkalaemia both overall and among patients sub-groups. The study protocol was reviewed and approved by the scientific review committee (SRC, reference number 13–030) for THIN [15].

## Methods

### Data source

A retrospective cohort study was performed using THIN. The database is representative of the UK population with regards to age, sex and geographic distribution, and has been validated for use in pharmacoepidemiological research [16, 17]. In the UK, the National Health Service provides free universal health care for all residents. Primary care practitioners (PCPs) are the first point of contact for patients, acting as a gatekeeper to secondary care; details of consultations in secondary care are communicated back to primary care and patients’ medical records are updated retrospectively. In addition, chronic disease in the UK is largely managed in the primary care setting, making THIN an excellent data source to evaluate the occurrence and risk factors for clinical outcomes in patients with heart failure.

### Study population

A previously identified cohort of patients aged 1–89 years between 1 January 2000 and 31 December 2005 with a first ever diagnosis of heart failure (inception cohort) and no previous diagnosis of cancer comprised our initial study cohort (*N* = 19,194); there were only 12 patients under 18 years old. Patients were required to have been registered for at least 1 year with their PCP and to have at least 1 year of computerized prescription history. At the time of diagnosis, approximately half (54.1 %) of the patients were ambulatory and managed by the PCP only, while 27.3 % were referred to a consultant and 18.6 % had a related hospitalization [18]. Among a random sample of the heart failure cohort (*n* = 200), 84 % had the diagnosis of incident heart failure validated by their PCP (*Ruigómez A, unpublished data*). All patients in the study cohort were followed from the start date (date of first recorded diagnosis of heart failure) until the earliest of the following: occurrence of hyperkalaemia, reaching 90 years of age, death, cancer or end of the study period (December 2011).

### Hyperkalaemia case ascertainment and definition

Potential cases of hyperkalaemia were identified through an initial computer search for abnormal serum K^+^ values or the Read codes ‘Hyperkalaemia’ and ‘Raised serum potassium level’ during follow-up. Guidelines define hyperkalaemia as a value of serum potassium (K^+^) >5.5 mmol [19, 20]. Recent clinical trials have therefore used fixed serum K^+^ thresholds to define hyperkalaemia, varying between ≥5.5 mmol/L and ≥6 mmol/L [7, 13, 21]. However, due to considerable differences in the upper bound of serum K^+^ normal range between practices contributing to THIN (range: 4.4 to 5.7 mmol/L), the former definition was not appropriate for our study. Thus, we tested several serum K^+^ thresholds to define hyperkalaemia, in order to analyze the absolute impact on estimates of outcome frequency and on the occurrence of a major clinical event (case-fatality). A summary of this sensitivity exercise is shown in Table 1.

**Table 1:** Number of HF patients with hyperkalaemia and incidence rates according to different hyperkalaemia definitions

Hyperkalemia definition	Patients identified with serum K^+^ qualifying value^a^	% over total cohort (N = 19,194)	IR per 100 person-years	30-day case fatality	1-year case fatality
Serum K^+^ >the upper limit^b^	5123	26.7	7.93 (7.72–8.14)	140 (2.7 %)	899 (17.5 %)
Serum K^+^ ≥5.5 mmol/L	3265	17.0	4.55 (4.40–4.71)	118 (3.6 %)	647 (19.8 %)
Serum K^+^ ≥10 % upper limit^b^	2155	11.2	2.87 (2.75–2.99)	78 (3.6 %)	460 (21.3 %)
Serum K^+^ >10 % upper limit^b^	1909	9.9	2.52 (2.41–2.63)	80 (4.2 %)	437 (22.9 %)
Serum K+ ≥6 mmol/L	1097	5.7	1.40 (1.32–1.49)	65 (5.9 %)	294 (26.8 %)
Serum K+ ≥20 % upper limit^b^	724	3.8	0.92 (0.85–0.98)	47 (6.5 %)	198 (27.3 %)

^a^Number of patients with a hyperkalaemia episode identified in the follow-up

^b^Upper bound of normal range used in the corresponding practice

*HF* heart failure, *IR* incidence rate

Exclusion criteria for all tested definitions were: serum K^+^ levels that returned to normal range within 3 days following the initial measurement that qualified as hyperkalaemia (possible pseudohyperkalaemia), and high values of serum K^+^ (≥10 mmol/L) considered as outliers. Of the six definitions tested, two were based on fixed absolute values irrespective of normal values applied in different laboratories; only 6 % of heart failure patients had values of K^+^ of ≥6 mmol/L, while 17 % had values equal or ≥5.5 mmol/L. In three definitions we used different proportional increases above the upper limit of the normal serum K^+^ value used by the respective reference laboratory, and for the remaining definition, any value above the upper range of normal bound for the corresponding practice was used to define hyperkalaemia (Table 1).

Among the six hyperkalaemia definitions tested, we decided to use a proportional increase above the referral laboratories’ upper bound of normal range and reject using a hyperkalaemia definition based on a unique absolute threshold (e.g. ≥5.5 mmol/L or ≥6 mmol/L) to define hyperkalaemia as in previously published studies [7, 8, 22]. Our decision was based on the results of our sensitivity analysis (Table 1), which showed considerable variation in hyperkalaemia incidence rates according to the definition used and because it accounted for the variation in the normal range of serum K^+^ values between different practice referral laboratories in THIN. The least restrictive definition of hyperkalaemia (value of serum K^+^ above the upper bound of normal value of the referral laboratory) classified more than a quarter of newly diagnosed heart failure patients as cases of hyperkalaemia, which might include non-clinically important cases, while the most stringent definition (≥20 % above the upper bound of normal range) classified the smallest percentage of heart failure patients as hyperkalaemia cases and could possibly have included only the most severe cases. Therefore, we chose to define an episode of hyperkalaemia as a recorded serum K^+^ value of ≥10 % above the upper bound of the normal range reported by the practice’s referral laboratory. In practice, our definition is approximately equivalent to adding 10 % above the range of 5–5.5 mmol/L, the upper limit of normal range in 96 % of practices in THIN. Additionally, in the absence of a recorded qualifying serum K^+^ value, patients with a recorded Read code for hyperkalaemia together with a referral to a specialist or hospital admission were also considered to be cases of hyperkalaemia in our final operational definition. This occurred in less than 1 % of our final set of hyperkalaemia cases. The date of first hyperkalaemia episode recorded during the follow-up was considered to be the outcome date.

### Data collection

Information on patient demographics, comorbidities, and healthcare utilization was extracted from THIN. Comorbidities included diabetes, renal impairment (identified using recorded creatinine values) and prior hyperkalaemia (identified using the same final operational definition described previously). We used the Modification of Diet in Renal Disease equation to calculate estimated glomerular filtration rate (eGFR) and to define renal impairment as eGFR <60 mL/min/1.73 m^2^. Healthcare utilization data comprised records of referrals and hospitalizations at the time of heart failure diagnosis.

### Statistical analysis

Using each of the six hyperkalaemia definitions, the incidence of hyperkalaemia was calculated as the number of incident cases per 100 person-years. Using our final selected operational definition of hyperkalaemia (serum K^+^ ≥10 % above upper bound of normal range), we estimated incidence rates with 95 % confidence intervals (CIs) both overall and stratified by age, sex, referral/hospitalization status at heart failure diagnosis (as a proxy for heart failure severity), diabetes, prior hyperkalaemia, and degree of renal impairment. Kaplan–Meier survival curves by age, sex, diabetes and renal function were produced and compared using the log-rank test. We also calculated 30-day and 1-year case-fatality rates using each of the six hyperkalaemia definitions. Statistical analyses were performed using Stata version 12.0 (StataCorp LP, College Station, TX, USA).

## Results and discussion

Among the cohort of 19,194 incident heart failure patients, 15,888 (83 %) patients had at least one laboratory value of serum K^+^ recorded after their first ever diagnosis of heart failure. Estimated incidence rates of hyperkalaemia varied from 0.92 to 7.93 per 100 person-years according to the definition of hyperkalaemia evaluated (Table 1). Applying our final operational definition of hyperkalaemia (K^+^ ≥10 % above upper bound of normal range), we identified 2,278 (12 %) patients with at least one qualifying value during follow-up. Among these, there were 38 patients with extremely high values that were considered computer entry errors, and 85 patients with a normal serum K^+^ value within 3 days following the first K^+^ measurement qualifying for hyperkalaemia (possible pseudohyperkalaemia); none of these patients were retained as cases of hyperkalaemia. We also identified 173 patients with a Read code for hyperkalaemia without a qualifying serum K^+^ value, but only 21 of these cases fulfilled the referral/hospitalization criteria required for inclusion as a hyperkalaemia case. In total, 2,176 patients (11.3 %) were considered to have an episode of hyperkalaemia over a mean follow-up period of 3.9 years (standard deviation ± 3.21 years), resulting in an overall incidence rate of 2.90 per 100 person-years, 95 % CI: 2.78–3.02. The mean age among cases of hyperkalaemia was 75 years (range: 23–89 years). For hyperkalaemia cases, short-term (30-day) and long-term (1-year) case-fatality rates increased within increasingly stringent hyperkalaemia definition. Short-term case-fatality ranged from 2.7 to 6.5 % while long-term case-fatality ranged from 17.5 to 27.3 %.

**Fig. 1:**
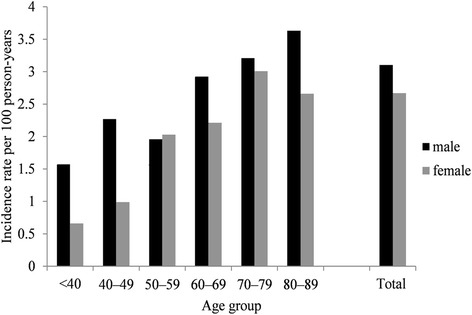
Incidence rate of hyperkalaemia among newly diagnosed heart failure patients by age and sex

Figure 1 shows the incidence rate of hyperkalaemia by age and sex strata. Overall incidence was slightly higher in men (3.10, 95 % CI: 2.93–3.27 per 100 person-years) than women (2.67, 95 % CI: 2.50–2.84 per 100 person-years), and increased with age. Over the entire follow-up period, 87.1 % of hyperkalaemia cases had five or more recorded serum K^+^ values after their heart failure diagnosis. Twenty percent of hyperkalaemia cases were ascertained (using a qualifying serum K^+^ value) within the first 6 months after the date of heart failure diagnosis. The number of recorded serum K^+^ values per patient, and the distribution of the time since heart failure diagnosis and the first recorded occurrence of hyperkalaemia, did not vary substantially according to whether the patient had diabetes or renal impairment.

**Fig. 2:**
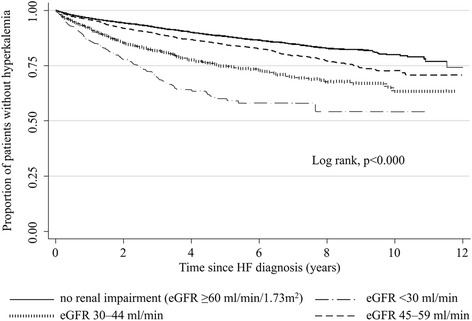
Proportion of heart failure patients not developing hyperkalaemia during follow-up according to the degree of renal impairment at heart failure diagnosis (Kaplan–Meier survival estimates). *eGFR* estimated glomerular filtration rate, *HF* heart failure

**Fig. 3:**
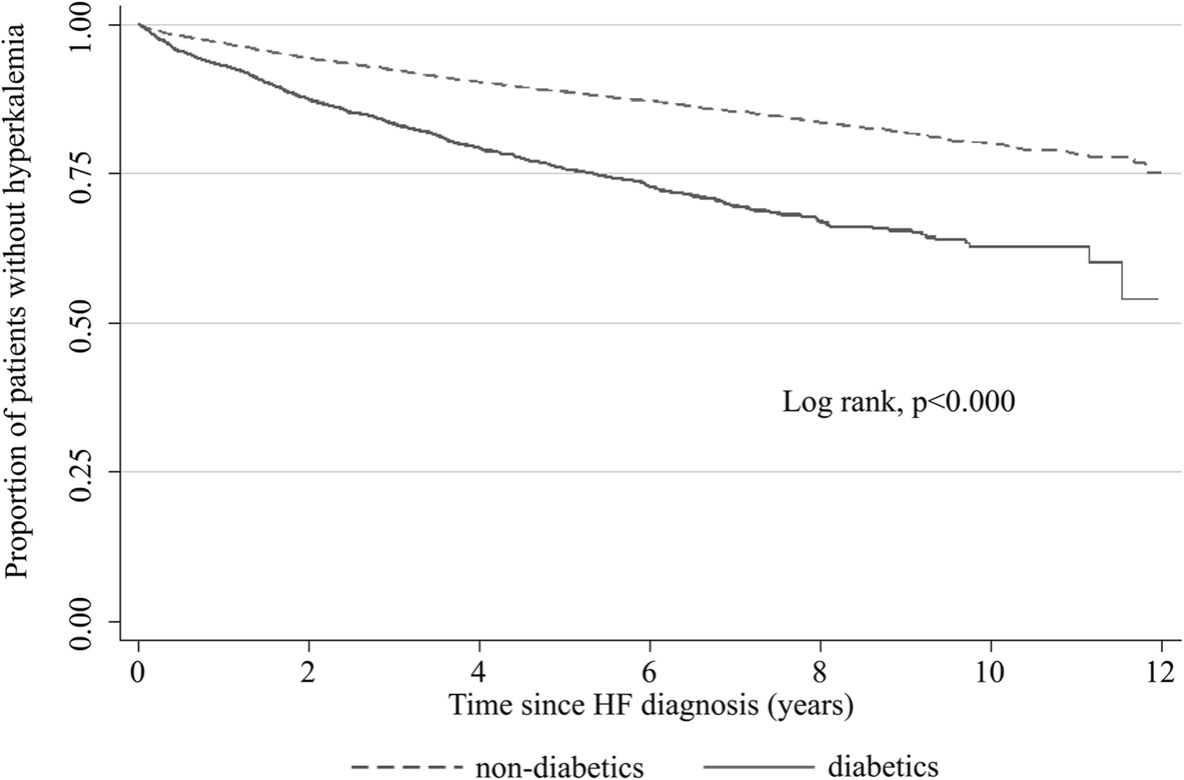
Proportion of heart failure patients not developing hyperkalaemia during follow-up according to diabetes status at heart failure diagnosis (Kaplan–Meier survival estimates). *HF* heart failure

When we examined the incidence of hyperkalaemia according to patient’s renal function, we found higher incidence rates with declining eGFR: 2.47 per 100 person-years for patients with normal renal function (eGFR ≥60 ml/min/1.73 m^2^) and 11.06 per 100 person-years for those with eGFR <30 ml/min/1.73 m^2^ (Table 2). Kaplan–Meier survival analysis showed the higher incidence among patients with renal impairment to be relatively constant over the duration of study (log-rank test, *p* <0.001) (Fig. 2). A higher incidence rate of hyperkalaemia was also found among patients with diabetes (5.50 per 100 person-years) compared with patients without diabetes (2.40 per 100 person-years) (Table 2). The higher incidence among diabetics was also relatively constant over the duration of the study (log-rank test, *p* <0.001) (Fig. 3). The incidence of hyperkalaemia also depended on heart failure severity at diagnosis when using hospitalization status at heart failure diagnosis as a rough proxy-measure. Nearly half (49.4 %) of the heart failure patients who developed hyperkalemia during follow-up were managed only by their PCP at the time of initial heart failure diagnosis, while the others were referred to a specialist (30.2 %) or were hospitalized (20.4 %) at initial diagnosis. Corresponding hyperkalaemia incidence rates (95 % CIs) during follow-up were 2.53 (2.38–2.68), 3.12 (2.89–3.37) and 3.84 (3.49–4.21), respectively.

**Table 2:** Incidence rate of hyperkalaemia in the HF cohort according to several potential risk factors

	Cases *N* = 2176	Person-years	IR per 100 person-years (95 % CI)
Sex			
Male	1259	40,639	3.10 (2.93–3.27)
Female	917	34,403	2.67 (2.50–2.84)
Age at HF diagnosis (years)			
20–49	49	2824	1.74 (1.31–2.30)
50–59	164	8355	1.96 (1.70–2.29)
60–69	500	17,836	2.80 (2.60–3.06)
70–79	961	30,329	3.17 (2.97–3.40)
≥80	502	15,698	3.20 (2.93–3.49)
Diabetes			
No	1507	62,887	2.40 (2.28–2.52)
Yes	669	12,155	5.50 (5.10–5.94)
Renal impairment			
No renal impairment (eGFR ≥60 ml/min/1.73^2^)	656	26,594	2.47 (2.29–2.66)
eGFR 45–59 ml/min/1.73^2^	497	14,392	3.45 (3.16–3.77)
eGFR 30–44 ml/min/1.73^2^	332	5503	6.03 (5.42–6.72)
eGFR <30 ml/min/1.73^2^	134	1211	11.06 (9.34–13.10)
No recorded eGFR	557	27,342	2.04 (1.87–2.21)
Hyperkalaemia prior to HF			
No	1946	73,237	2.66 (2.54–2.78)
Yes	230	1805	12.74 (11.20–14.50)
Within 90 days before	51	363	14.05 (10.68–18.48)
Within 91–365 days before	84	511	16.45 (13.28–20.37)
Within >365 days before	95	931	10.20 (8.34–12.47)

*CI* confidence interval, *eGFR* estimated glomerular filtration rate, *HF* heart failure, *PCP* primary care practitioner

^a^Includes the 2155 cases of hyperkalaemia identified based on having recorded serum K^+^ ≥10 % upper limit of normal and a further 21 cases of hyperkalaemia identified using Read codes only but who also had a record of a referral or hospitalization

Few studies have evaluated the incidence of hyperkalaemia in clinical practice. In our large population-based cohort study in the UK, the incidence rate of hyperkalaemia among newly diagnosed heart failure patients in real-life clinical practice was found to be 2.90 per 100 person-years, using a definition of serum K^+^ of ≥10 % above the upper limit of normal range. Our study also has the novelty of investigating the effects of differing diagnostic thresholds on the resulting incidence rates in this patient population, highlighting the importance of applying a precise definition of the outcome. We found that the K^+^ threshold used to define hyperkalaemia had a significant impact on incidence rate estimates – an almost 10-fold difference was observed between the most restrictive and the most liberal operational definition used (range: 0.92–7.93 per 100 person-years). We also showed increasing case-fatality rates with increasingly more stringent hyperkalaemia definitions. In previous clinical studies, hyperkalaemia has been defined as any value of serum K^+^ above an absolute value. These studies have generally involved a particular referral laboratory applying an absolute serum K^+^ value to define hyperkalaemia [21, 22]. However, this definition is not the most appropriate for studies of patients coming from different general practices, which use different referral laboratories with their own normal range of laboratory parameters, as in our study cohort, and we considered this factor very meaningful when deciding upon our final operational definition. Also, there is no consensus concerning the serum K^+^ threshold to identify hyperkalaemia cases, making it difficult to compare reported incidence rates between studies. The most commonly used threshold to define hyperkalaemia is a serum K^+^ value of >5.5–6.0 mmol/L [6–8, 13, 21, 23, 24]. Other studies have used a lower serum K^+^ threshold, such as 5.0 mmol/l [9, 10] or 5.3 mmol/l [11], while some have not used a specific serum K^+^ threshold, but used what the investigator considered to be an important rise in serum K^+^ level based on clinical criteria [12]. Furthermore, other researchers have defined hyperkalaemia based only on International Classification of Diseases codes either in the general practice setting [25] or when diagnosed upon hospital admission [2, 26]. In our study, we retained only 21 patients (1 %) as cases of hyperkalaemia based only on Read codes but we further required them to have been referred or hospitalized at that point.

Clinical manifestations of hyperkalaemia do not usually occur at serum K^+^ values <5.5 mmol/L with severe hyperkalaemia usually considered as serum K^+^ ≥6.0 mmol/L [24]. The risk of potential hazards increase with increasing serum K^+^ levels, with risks especially increased when serum K^+^ levels are >7.5 mmol/L [6, 23]. Limiting case definitions to include only severe cases of hyperkalaemia (≥6 mmol/L) has been shown to significantly underestimate the risk of hyperkalaemia [13]. Furthermore, available evidence regarding the risk of hyperkalaemia in patients with heart failure comes mainly from clinical trials investigating the efficacy of RAAS antagonists as heart failure treatment, with reported proportions ranging from 1.1 % when thresholds are more restrictive [13] up to 13.4 % with less restrictive thresholds [12, 13, 21]. It is also difficult to compare rates between studies due to differences in the baseline characteristics of the patients enrolled (e.g. age, severity of heart failure, concomitant medications and comorbidities). Patients in clinical trials are also selected carefully, specifically excluding those at a higher risk of experiencing potential adverse events, and with intensive monitoring during follow-up. Estimates from clinical trial settings might not be comparable to those in clinical practice where estimates as high as 24 and 36 % have been reported, albeit from small study populations [3, 10]. Our findings are in line with those from a recent multisite database study conducted by Raebel et al. [27] involving ambulatory patients with diabetes newly initiating a therapy with angiotensin-converting enzyme (ACE) inhibitors, angiotensin receptor blockers (ARBs) or spironolactone. The authors found that the degree of restriction in hyperkalaemia definitions affected incidence rates in a similar way as in our study. In a retrospective analysis of data in the Studies of Left Ventricular Dysfunction trials, de Denus et al. [13] also showed the incidence of hyperkalaemia to vary substantially depending on the serum K^+^ thresholds used to define a hyperkalaemia episode.

Monitoring of serum K^+^ levels in patients with heart failure is critically important to prevent severe outcomes, especially among patients at high risk. In line with previous studies that have documented advanced age [9, 12], diabetes [9, 12, 13], renal failure [9, 12, 13, 21], and having prior high K^+^ values [12, 13, 21] to be significant predictors of hyperkalaemia, we found hyperkalaemia incidence rates to be higher among these patient populations. The link between renal failure and diabetes with hyperkalaemia in patients with heart failure is particularly well established and we found the risk in diabetics to be relatively constant over the duration of follow-up. Heart failure patients are particularly vulnerable to the development of hyperkalaemia when potassium excretion is already impaired due to disease-related decline in glomerular filtration, or circumstances, such as in diabetes, when aldosterone production is decreased [4]. Furthermore, most of these patients are on medications which block the RAAS, such as ACE inhibitors, ARBs or aldosterone antagonists. A comprehensive evaluation of hyperkalaemia risk factors, including medications, has been published recently [28].

Our study followed up a large cohort of patients from a validated primary care database representative of the UK population for up to 12 years [16]. It is based on real-life data, reflecting actual practice in the primary care setting as opposed to clinical trials where patients are closely monitored for adverse events, such as hyperkalaemia. Another strength of our study is the high proportion of cases (99 %) that were ascertained using an operational definition of hyperkalaemia based on laboratory data (values of serum K^+^) thereby decreasing misclassification in the assessment of hyperkalaemia episodes compared with assessment based only on diagnostic codes [27]. Our study also has limitations. It is possible that there was some under-ascertainment of hyperkalaemia cases because the level of detection is largely dependent on the level of awareness and monitoring by the PCP. Also, although hyperkalaemia is a condition largely managed in primary care, more severe cases are hospitalized and we would rely on this information to be communicated back to the PCP and recorded in the patient’s medical notes. On the other hand, some selection bias towards more severe patients may have occurred because patients with greater comorbidity are more likely to be investigated. The impact of any such potential selection bias, however, is likely to be small because the majority of heart failure patients (over 80 %) had at least one laboratory value of serum K^+^ recorded.

## Conclusions

To properly ascertain the incidence of hyperkalaemia in patients with heart failure and thereby evaluate hyperkalaemia risk and guide therapeutic decision making, it is important that a precise definition of the clinical event is used. In addition, when different referral laboratories are used in research studies, such as in primary care studies, we propose that a proportional increase above the upper bound of normal range in the corresponding laboratory is used instead of a fixed absolute K^+^ value. Careful monitoring of serum K^+^ levels, particularly in more elderly patients and in those with prior comorbidity such as diabetes or renal impairment is recommended.

## Declarations

### Ethics and consent to participate

Ethical approval was provided by the scientific review committee (SRC, reference number 13-030) for THIN. This study involved the use of unlinked anonymized patient medical records, which contained no information that could reasonably be used, by anyone, to identify people.

### Consent to publish

Not applicable.

### Availability of data and materials

The data supporting our findings is held at The Spanish Centre for Pharmacepidemiologic Research (CEIFE), Madrid, Spain and can be shared upon contacting the corresponding author.

## Abbreviations

CI: confidence interval
EMRs: electronic medical records
eGFR: estimated glomerular filtration rate
HF: heart failure
IR: incidence rate
K^+^: serum potassium
PCP: primary care practitioner
RAAS: renin-angiotensin-aldosterone system
THIN: The Health Improvement Network
UK: United Kingdom
